# A case series of calcium pyrophosphate deposition in the spine: An underrecognized debilitating disease

**DOI:** 10.1093/jnen/nlaf151

**Published:** 2025-12-23

**Authors:** Serica J Hallstead, Jennifer M Dailey, Michael Punsoni

**Affiliations:** Department of Neuropathology and Laboratory Medicine, Rhode Island Hospital and Warren Alpert Medical School at Brown University, Providence, RI, United States; Department of Neuropathology and Laboratory Medicine, Rhode Island Hospital and Warren Alpert Medical School at Brown University, Providence, RI, United States; Department of Neuropathology and Laboratory Medicine, Rhode Island Hospital and Warren Alpert Medical School at Brown University, Providence, RI, United States

**Keywords:** calcium pyrophosphate, CPPD, lumbar, pseudogout, spine, thoracic

## Abstract

Calcium pyrophosphate deposition (CPPD) disease is an inflammatory arthritis prevalent in elderly individuals. Spinal CPPD is uncommon, and presentation can vary widely with common clinical and radiographic mimics. We identified 9 cases of lumbar and 1 case of thoracic spinal CPPD (age range: 63-85 years; 5 male, 5 female) at our institution over a 10-year period. All presented with pain (90% chronic, 10% acute); 4 had prior instrumentation and 1 had prior trauma. CPPD was not clinically suspected in any case. Preoperative imaging diagnoses include central canal stenosis, neuroforaminal narrowing, septic arthropathy, synovial cyst, degeneration, and epidural mass/abscess. Intraoperatively, a spinal cyst was most often described. All pathologic examinations revealed purple granular calcified material with positively birefringent rhomboid crystals embedded within fibrocartilage. Following a neuropathologic diagnosis, only 2 patients received CPPD treatment. This study aims to highlight that spinal CPPD is underdiagnosed and undertreated. Although clinical presentations are variable, wider recognition of this rare disease among neuropathologists receiving spinal specimens may provide earlier diagnosis. Accurate pathologic diagnosis can improve identification, refine clinical suspicion, and facilitate management. Improved identification, particularly in elderly patients and those with prior trauma/instrumentation, will promote appropriate therapy and management.

## INTRODUCTION

Calcium pyrophosphate deposition (CPPD) disease is an inflammatory arthritis that affects 4%-7% of adults, particularly of advanced age or with prior joint trauma or instrumentation in Europe and the United States.^1^ The most common risk factor for CPPD is age, with the highest incidence observed in patients 60 years or older.^2^ This arthropathy can present in several ways. The patient may be asymptomatic, present acutely as pseudogout or present chronically as an inflammatory arthritis that mimics other rheumatologic diseases, such as pseudo-polymyalgia rheumatica, pseudo-neuropathic arthropathy, and tophaceous CPPD.^3^ The most common co-morbidity with CPPD is osteoarthritis; the diagnosis can be especially challenging in patients who have both conditions.^2^ Although nonspecific, the most common radiographic finding in CPPD is chondrocalcinosis, ie calcification of joint cartilage.^2^ The gold standard diagnostic method is the identification of calcium pyrophosphate (CPP) crystals in aspirated synovial fluid via plain and polarized light microscopy. Calcium pyrophosphate crystals are more difficult to visualize compared to the monosodium urate (MSU) crystals of gout and exhibit weaker positive birefringence. Currently, there are no medical therapies capable of dissolving CPP crystals; therefore, the mainstay of treatment includes controlling inflammation and pain.^2^ Unfortunately, due to a paucity of randomized controlled trials and the lack of disease modifying treatments, in rare cases of CPPD diagnosis, patients are often treated with agents used for gout including non-steroidal anti-inflammatories (NSAIDs), glucocorticoids, and colchicine.^1^

CPPD most often affects the knee and wrist joints but a limited number of reports also describe CPPD of the spine. Cervical spine involvement is well characterized in the literature as “crowded dens syndrome” and represents approximately 5% of acute presentations.^2^ In contrast, a majority of articles describing lumbar CPPD are case reports with only a few large studies.

The more comprehensive studies include literature reviews and case series and highlight axial CPPD as a mimic that masquerades as spinal infections and compressive cystic masses; this results in potential overuse of antibiotics and unnecessary invasive intervention.^4^ Resnick and Pineda compared radiographs and pathology to conclude that structural changes of the spine in patients with axial CPPD may resemble degenerative arthritis and that osseous changes caused by crystal induced disc changes mimic infection, neuroarthropathy, and progressive scoliosis on imaging.^5^ Gruber et al reviewed 211 intervertebral disc specimens to determine the incidence of crystals and discovered that 14.7% had crystals compatible with calcium pyrophosphate, highlighting the need for consideration of this pathologic finding, the under recognition of this disease process and the possible role of crystal formation in the spine.^6^

CPPD may be considered to be encompassed by the larger umbrella of degenerative disc disease as it is often accompanied by a constellation of histologic features characteristic of degenerative changes. These features include neovascularization of disc tissue, synovial proliferation, chondrocyte proliferation, structural alterations, granular changes, and mucous degeneration.^7^^,^^8^ Some authors have employed a histologic degeneration score in their investigations into degenerative disc disease. Weiler et al^8^ modified a classification originally proposed by Boos et al^9^ in 2002 to explore the correlation between severity of degeneration and various demographic and biologic factors. They graded the degree of proliferation of chondrocyte clusters, as well as the presence of tears, amorphous eosinophilic granules, and mucopolysaccharide deposition within the fibrocartilaginous disc matrix. Calcium pyrophosphate dehydrate deposits are not specifically included within the aforementioned histologic degeneration score; however, Pytel et al described crystal deposition in approximately 3% of extradural spinal specimens excised for degenerative spine disease within their study, which correlates closely with our investigation.^7^

Gupta et al reviewed several case reports and described an additional case of a patient who presented with worsening back pain and imaging findings concerning for infection.^10^ Biopsy cultures were negative and the patient improved with the administration of prednisone. Overall, the authors emphasized the importance of correlating imaging data with pathology results especially when the clinical suspicion of CPPD is high and infectious etiologies are ruled out to avoid invasive or unnecessary treatments in elderly patients with acute back pain. In a case series by Moshrif et al, the investigators determined that 19 of 152 patients (12.5%) hospitalized with CPPD had lumbar involvement, which is similar to the findings by Gruber et al.^11^ Out of these 19 patients, 5 had prior spinal instrumentation and 4 had suspected lumbar infections.

From 2014 and 2023, several case reports of lumbar CPPD were published; the majority described an infectious etiology as the leading clinical differential prior to microscopic tissue evaluation by neuropathology (Table 1). Based on the available literature, the hallmark of diagnostic uncertainty in axial, particularly lumbar, CPPD cases is its mimicry of other conditions, primarily spinal infections. This study aimed to review cases at one institution where CPPD was not included in the preoperative differential diagnosis to further characterize the clinical presentation of CPPD, to identify clinical mimics, to describe in detail the neuropathologic characteristics of CPPD in the spine and to provide recommendations for improved recognition of this debilitating disease.

**Table 1.** Summary of calcium pyrophosphate deposition disease case reports.

Authors	Patient demographics	Clinical suspicion
Grobost et al (2014)^12^	85-year-old male	Septic spondylodiscitis
Bridges et al (2017)^13^	66-year-old female status post lumbar fusion	Discitis and epidural abscess
Loizidis et al (2019)^14^	55-year-old female	Osteomyelitis and discitis
Mazzoni et al (2018)^15^	68-year-old female	Facet joint septic arthritis
Greca et al (2020)^16^	80-year-old female with prior spinal trauma	Osteomyelitis and discitis
Gupta et al (2023)^10^	61-year-old male	Osteomyelitis and discitis
Ni and Kumar (2023)^17^	75-year-old male status post fusion	Spinal neuroarthropathy

This list demonstrates the tendency of CPPD to mimic other spinal pathologies, especially infection. All patients were subsequently diagnosed with CPPD. CPPD: calcium pyrophosphate deposition.

## METHODS

A 10-year retrospective review, from January of 2014 to January of 2024, was performed at Rhode Island Hospital that identified 296 intervertebral disc specimens. A natural language search was then conducted in CoPath Laboratory Information System using search terms “calcium phosphate,” “pseudogout,” “CPPD,” “spine,” and “lumbar.” Eight recent cases were identified between 2022 and 2024 while 2 remote cases were from 2015 to 2020 for a total of 10 axial CPPD cases (1 thoracic and 9 lumbar). Neuropathology reports and the electronic medical record were reviewed. All 10 patients received a pathological diagnosis compatible with CPPD, although exact verbiage in the reports varied. Patient demographics (eg age and biological sex) were recorded. Histories and physical examinations provided information on symptom onset and specific clinical presentation, as well as history of prior spinal trauma or instrumentation and any known spinal degenerative diseases. The available histories did not consistently include a precise description regarding timing of patient presentation; therefore, the authors classified onset into 4 general categories: acute (days to weeks), subacute (1 month to 3 months), chronic (greater than 3 months), and acute on chronic (new symptoms or abrupt worsening in a patient with prior history). Preoperative diagnoses, procedure names, and operative findings were reviewed. Patient outcomes were investigated regarding rheumatologic workup or treatment for CPPD upon receipt of pathology results, administration of antibiotics, additional spinal surgeries, and overall morbidity. A poor or favorable outcome was assigned to each patient based on post-operative morbidity, including complications and necessitation of additional intervention, including surgery. No exclusion criteria were identified, and all 10 patients resulting from the CoPath natural language search between 2014 and 2024 were included. Patients were designated as #1 through #10 chronologically by procedure date.

## RESULTS

**Figure 1.**
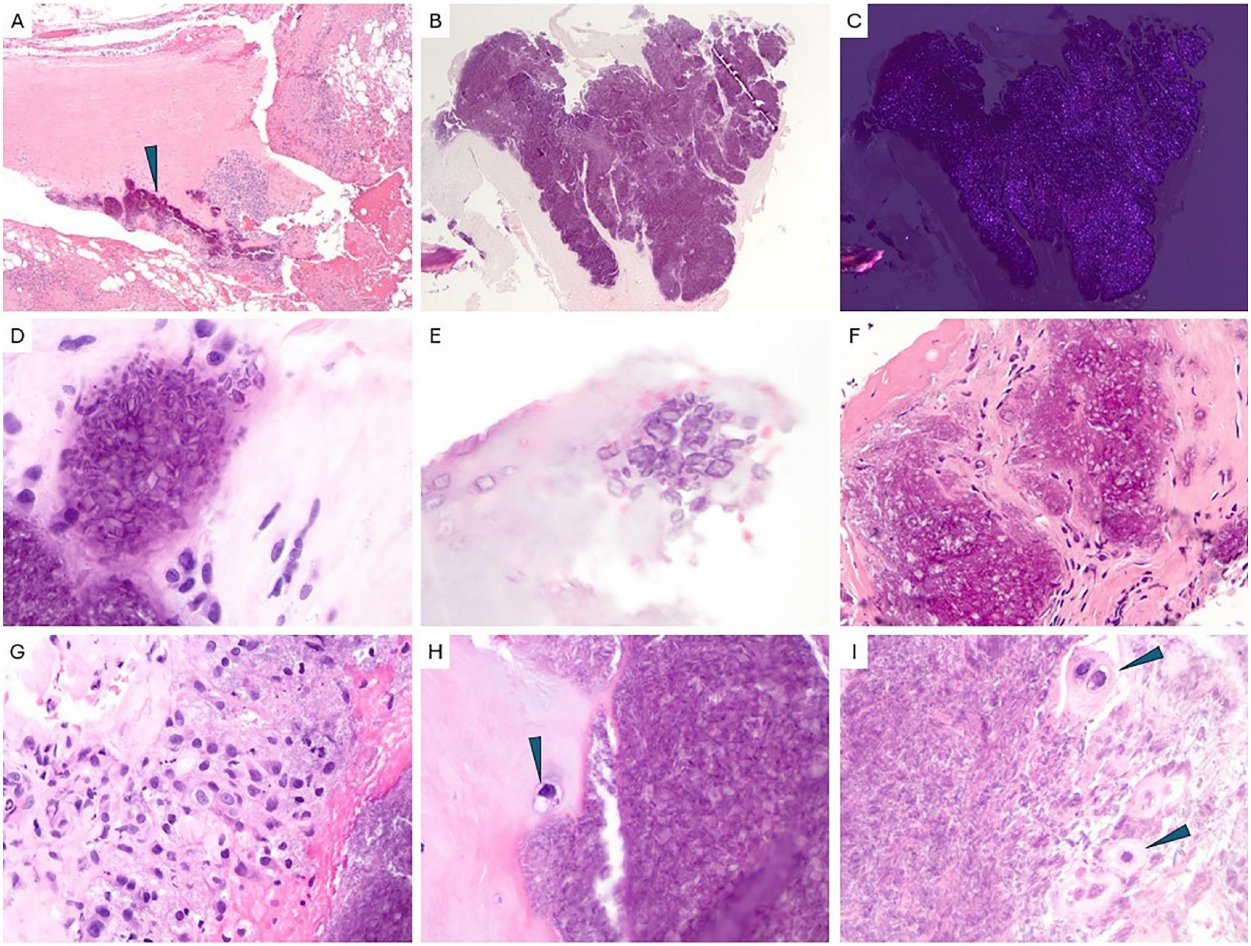
Histopathology of CPPD. (A) Low-power image of dense fibroconnective tissue with well-delineated nodular deposits of calcified basophilic material (arrowhead) with inflammation and granulation tissue (20×). (B, C) Extensive deposition of basophilic material that shows birefringence with polarization (40×). (D) Rhomboid crystalline aggregates within the basophilic calcified material and chondroid metaplasia (600×). (E) Loose aggregate of rhomboid shaped crystals with a myxoid background (600×). (F) Aggregates of basophilic material with rhomboid crystals and surrounding chronic inflammation and chondrocyte metaplasia. (G) Calcium pyrophosphate crystals surrounded by a strong histiocytic reaction and metaplastic and hypertrophic chondroid cells (400×). (H, I) Chondrocyte metaplasia and hyperplasia (600×). Hematoxylin and eosin.

The pathological features of CPPD in the spine are similar to morphology observed in tissue from other sites in the body. Examination of all spinal tissue from our cohort revealed scattered aggregates of granular, basophilic calcified material embedded within dense collagen-rich fibroconnective tissue (Figure 1A), most likely representing ligamentum flavum. The basophilic material was admixed with positively birefringent rhomboid-shaped crystalline structures (Figure 1B and C) with occasional needle-shaped forms when subjected to standard polarizing light microscopy. These rhomboid crystals, which represent perhaps the most remarkable feature of this disease, appear bilaterally symmetrical, and diamond shaped at high power (Figure 1D and E). A subset of cases showed chronic inflammation (Figure 1F), a prominent histiocytic reaction (Figure 1G), stromal hyalinization (Figure 1G), with metaplastic and hypertrophic chondrocyte morphology, occasionally with a myxoid background (Figure 1H). These characteristic features were often accompanied by degenerative and reactive changes in fibrocartilaginous tissue (occasional clefts in the tissue, chondroid clusters and loose chondroid matrix, neovascularization, dystrophic calcifications, and mucoid stromal changes).

**Figure 2.**
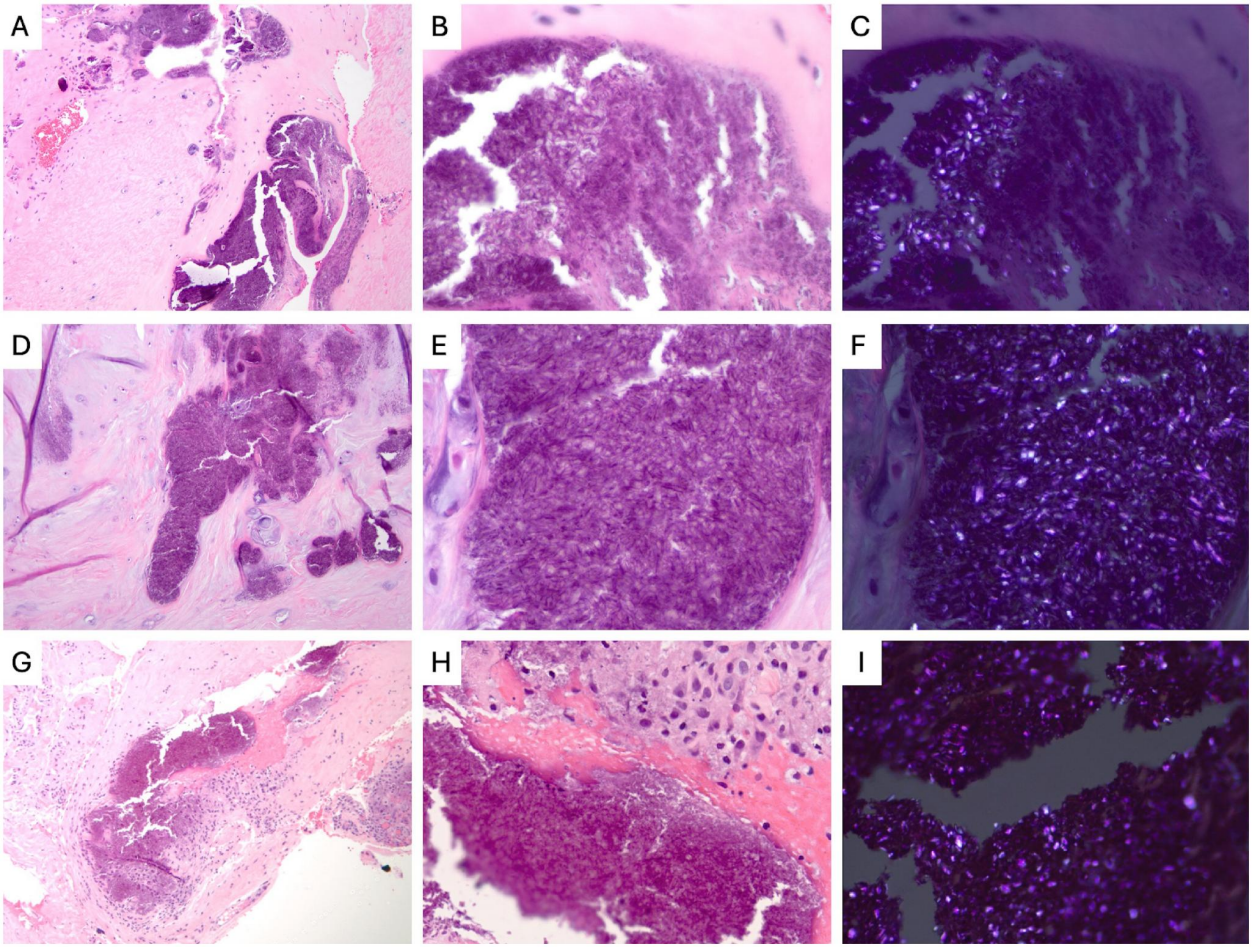
Representative images of CPPD histopathology in patients with distinct clinical presentations (acute, chronic, and acute on chronic). (A-C) Acute presentation: Spinal histopathology shows dense fibroconnective tissue which contains small, nodular basophilic aggregates surrounded by a paucicellular histiocytic response and metaplastic chondrocytes. Rare foci of hemorrhage are noted. The basophilic aggregates contain numerous rhomboid-shaped crystals, which are positively birefringent under polarized light. (A, 100×, B-C, 600×). (D-F) Chronic presentation: Spinal histopathology shows fibroconnective tissue with prominent stromal myxoid degeneration, scattered well-formed basophilic aggregates with rhomboid crystals, granulation tissue, macrophages, and chondrocyte metaplasia. The aggregates contain rhomboid shaped crystals with positive birefringence. (D, 100×, E-F, 600×). (G-I) Acute on chronic presentation: Spinal histopathology shows loosely formed basophilic aggregates of calcified material containing positively birefringent rhomboid shaped crystals with a prominent histiocytic response and adjacent areas of granulation tissue. (G, 100×, H, 400×, I, 600×). Hematoxylin and eosin.

Although the patients in this series share the same general pathologic features of CPPD, we examined tissue from cases in patients with acute, chronic, and acute on chronic presentations (Figure 2). Tissue from the patient with an acute presentation showed small, nodular aggregates of material with a paucicellular histiocytic response and metaplastic chondrocytes surrounding the aggregates (Figure 2A-C). Of the patients with chronic presentation, there was chondrocyte metaplasia with a prominent myxoid background favored to represent a degenerative change (Figure 2D-F). The patient with an acute on chronic presentation showed areas of stromal hyalinization, granulation tissue, and a strong histiocytic response with loosely formed aggregates (Figure 2G-I). All cases showed positively birefringent rhomboid-shaped crystalline deposits that appear bright blue-white under polarized light, typical of calcium pyrophosphate dihydrate crystals (Figure 2C, F, and I).

Following a review of 296 intervertebral disc specimens over a period of 10 years, 10 patients (approximately 3%), who were ultimately diagnosed with CPPD, were included in this study (*n* = 10; 5 biologic males, 5 biologic females) ranging in age from 63 to 85 years (mean, SD = 73.8 years, 8.13). Patients either presented, more often, with months to years of chronic back and/or leg pain, or less often with an acute-on-chronic presentation. Of the 2 patients who underwent a spinal procedure prior to an acute presentation. Only 1 patient received a facet joint injection, while the other patient had multiple prior revisions of spinal hardware. One patient presented acutely, and 2 had a subacute presentation. Other symptoms included paresthesias (eg leg numbness, weakness, tingling, spasticity, foot numbness, weakness, and tingling), an increased frequency of falls, difficulty standing and ambulating, and urinary incontinence. Four patients had prior spinal instrumentation, including one with remote spinal trauma from a motor vehicle accident. Two patients had elevated inflammatory markers at presentation and one of those patients had a fever (Table 2).

**Table 2.** Patient characteristics, presentations, and preoperative diagnoses.

Cohort Characteristics, *n* = 10						
Age at presentation, mean (SD) 73.8 years (8.13) Biological sex 5 males and 5 females						
ID	Age (y)	Sex	Time course	Presentation	Relevant history	Preoperative diagnosis
1	69	F	Acute on chronic	Chronic left LE pain with sudden worsening, impaired standing and ambulation following facet injection	RA, OA, FM, SLE, DDD, prior spinal instrumentation with C2-T2 fusion, MRSA infections, multiple I&Ds, hardware removal	Lumbar radiculopathy
2	67	M	Acute	Lower back pain with right LE numbness and foot drop	Traumatic thoracic myelopathy from an MVA, prior lumbar laminectomy	Lumbar radiculopathy and synovial cyst
3	79	M	Chronic	Back and left LE pain	OA, scoliosis, prostate cancer s/p prostatectomy	Lumbar radiculopathy, L5-S1 spondylolisthesis
4	75	M	Chronic	Back and bilateral LE pain with left LE numbness	N/A	Lumbar spondylosis and stenosis with neurogenic claudication
5	71	M	Acute on chronic	Acute worsening of years long lower back and LE pain, difficulty ambulating, fever, elevated inflammatory markers	N/A	Lumbar stenosis with neurogenic claudication
6	82	M	Subacute	Lower back pain, LE pain, spasticity, weakness, falls	Gout on allopurinol, PD, RA	Lumbar epidural mass
7	83	F	Subacute	Bilateral LE weakness, numbness, difficulty standing/ambulating, urinary incontinence	N/A	Lumbar disc herniation
8	64	F	Acute on Chronic	Post-operative LE weakness and minimal movement, elevated inflammatory markers	Adolescent idiopathic scoliosis, chronic back pain and spinal instrumentation	Thoracic epidural abscess
9	63	F	Chronic	Lower back pain with radiation to bilateral LEs, numbness, weakness, and tingling	Prior L4-S1 laminectomy, Lyme Disease, OA, Poland Syndrome, Scoliosis	Lumbar spondylosis and stenosis with radiculopathy, synovial cyst
10	85	F	Chronic	Lower back pain with radiation to posterior buttocks and LEs, difficulty standing/ambulating	OA, MGUS	Lumbar spinal stenosis with neurogenic claudication

Abbreviations: C, cervical; DDD, degenerative disc disease; FM, Fibromyalgia; LE, lower extremities; MGUS, monoclonal gammopathy of unknown significance; MRSA, methicillin-resistant *Staphylococcus aureus*; MVA, motor vehicle accident; N/A, not applicable; OA, osteoarthritis; PD, Parkinson’s Disease; RA, rheumatoid arthritis; S, sacral; SLE, systemic lupus erythematosus; y, years.

The most common imaging findings were central canal stenosis (8 patients) and neuroforaminal narrowing (5 patients). The most common corresponding preoperative diagnoses were neurogenic claudication and radiculopathy. Preoperative imaging showed evidence of degeneration in a small subset of patients (#3, #4, and #9), while an epidural mass or abscess was suspected in 2 patients (#6 and #8), and a synovial cyst (2 patients) in an additional 2. Of note, the radiological findings for 1 patient raised concern for septic facet arthropathy with a dorsal epidural phlegmon, but the preoperative clinical diagnosis was lumbar stenosis with neurogenic claudication. A synovial cyst was found on imaging for 1 patient but was not included in the preoperative diagnosis (Tables 2 and 3). An additional 2 patients were found to have a synovial cyst intraoperatively that was not reported on imaging. Other intraoperative findings included degenerative disc material, calcified disc material, an extradural facet cyst, an epidural cyst, ligamentum flavum hypertrophy, and inflammatory material (Table 3).

**Table 3.** Patient imaging findings by radiologist read and procedures with operative findings.

Patient	Imaging findings	Procedure	Operative findings
1	CT: Lucent lesion within L3 lamina on MRI: T1w/T2w/STIR hyperintense, homogenously enhancing	L4-5 discectomy with foraminotomy; L3 lamina and cyst excision	L3 fibrogelatinous lesion consistent with DDD or synovial cyst
2	L4-L5 synovial cyst	L4-L5 laminoforaminotomy and synovial cyst resection	L4-L5 synovial cyst
3	DDD and facet arthropathy most pronounced at L4-L5	L3-S1 posterior lumbar decompression and fusion	L4 dorsal extradural facet cyst
4	L4-5, L5-S1 disc bulge and central canal stenosis secondary to ligamentum flavum hypertrophy and herniation, bilateral neuroforaminal stenosis, likely degenerative	L4-L5 laminectomy, discectomy	L4-5 severe stenosis with partially calcified disc material
5	L4-5 effusion of right facet joint with features favoring septic facet arthropathy with dorsal epidural phlegmon	Posterior lumbar decompression	Ligamentum flavum hypertrophy with inflammatory material and overgrown facets
6	L2/3 dorsal epidural mass contributing to severe central canal stenosis	L2-3 laminectomy for excision of epidural lesion other than neoplasm	Resection of ligamentum flavum with “mass,” consistent with a large, extruded disc
7	L4/5 anterolisthesis, facet arthrosis, bilateral foraminal narrowing	Right L4-5 decompression	Large L4-5 synovial cyst, epidural cyst
8	Small epidural fluid collection from C4 to T4, central canal stenosis from C5-6 to T2-3 secondary to spondylosis	T3 laminectomy and decompression	Epidural fat, no obvious phlegmon
9	Severe central canal stenosis at L3-L4 with synovial cyst, multilevel DDD	L3/4 laminectomy for decompression, resection of synovial cyst	Right L3 synovial cyst
10	L4-5 facet arthropathy with critical spinal stenosis, ligamentum flavum hypertrophy, synovial cyst in dorsal epidural space	Posterior L4-5, L5-S1 microdecompression	Thickened ligamentum flavum and synovial cyst

Abbreviations: CT, computed tomography; DDD, degenerative disc disease; MRI, magnetic resonance imaging; T1w, T1-weighted; T2w, T2-weighted; STIR, short tau inversion recovery; C, cervical; T, thoracic; L, lumbar; S, sacral.

CPPD was not included in the differential diagnosis of any of the cases; however, pathologic examinations revealed polarizing rhomboid crystalline deposits, consistent with CPPD in all patients. The most common anatomical site of pathology involved L4-L5 of the lumbar spine in 7 patients and L2-L3 in 2 patients. One patient had thoracic pathology at T3.

Eighty percent of patients had a favorable outcome following surgery with eventual symptom resolution but 2 patients had poor outcomes; in those cases, 1 patient required numerous additional spinal surgeries with minimal symptom resolution and another patient was paraplegic post-operatively. Four of 10 patients were given post-operative antibiotics to reduce the likelihood of post-operative infection.

Only 2 patients received a referral to rheumatology for a full CPPD work up immediately following the pathologic diagnosis. Subsequent screening of one such patient revealed a 30-year history of joint pain and swelling prior to presentation that rheumatology favored to be consistent with polyarticular pseudogout. The patient was later started on methotrexate. Rheumatology recommended imaging studies for a second patient of the knees and wrists that revealed chondrocalcinosis. The patient was subsequently started on a prednisone taper and colchicine. Interestingly, 4 years after the neuropathologic diagnosis of CPPD was made in the lumbar spine of 1 patient, and following multiple additional spinal surgeries, CPP crystals were found in a wrist joint aspirate, which prompted evaluation for CPPD (Table 4).

**Table 4.** Patient outcomes: medical management,^a^ additional surgeries, and complications.

ID	**Outcome** ^b^	CPPD medical therapy	Antibiotics	Additional spinal surgery	Complications
1	Poor	Colchicine^c^	Yes, culture (+)	Eight	None
2	Favorable	None	None	None	None
3	Favorable	None	None	None	None
4	Favorable	None	None	None	None
5	Favorable	Methotrexate^c^	Yes, culture (−)	None	None
6	Favorable	None	None	None	None
7	Favorable	None	Yes, for UTI	None	None
8	Poor	Prednisone and colchicine^c^	Yes, cultures unknown	None	LE paraplegia
9	Favorable	None	None	None	None
10	Favorable	None	None	None	None

Abbreviations: y, years; LE, lower extremity; UTI, urinary tract infection; (+), positive; (-), negative.

a Medical management includes any CPPD medical therapy or post-operative antibiotics.

b The designation of poor versus favorable outcome was decided based on if the patient required additional spinal surgeries and any significant post-operative complications.

c Patients referred to Rheumatology for further medical management.

## DISCUSSION

CPPD disease of the lumbar spine is underdiagnosed and therefore undertreated. In this study, we reviewed 10 cases from our institution to further characterize the diverse clinical presentation of CPPD and to highlight the critical role that accurate and comprehensive pathologic diagnosis plays in these cases.

Biologic sex was equally distributed in our cohort, with a mean age of 74 years at presentation and ranging from 63 to 85 years. Pathology was located in the lumbar spine in 90% of cases, with one case in the thoracic spine. The majority (70%) of patients had chronic symptoms, with their onset characterized as either chronic or acute-on-chronic. Of the remaining cases (30%) only 1 patient presented acutely, and the remaining 2 had subacute presentations. Back pain was the most frequent presenting symptom (in 80% of patients). The other 20% of patients presented with leg symptoms, either pain or weakness.

Radiculopathy or claudication was the preoperative diagnosis in most patients. In the remaining 30% of patients, an epidural mass, epidural abscess, or disc herniation was suspected. A synovial cyst was found in half of the patients intraoperatively, but this finding was only included in the preoperative diagnosis of 2 patients.

Crystal arthropathy was not suspected in any of our patients, and despite pathological diagnosis, only 2 patients received post-operative work up and treatment for CPPD at the time of reporting. This may be due to a variety of reasons including the lack of specific clinical symptoms for CPPD in combination with its extreme rarity in the axial skeleton, especially the lumbar spine, the fact that CPPD may have a highly variable presentation, and the complex nature of back pain that leads to a wide differential diagnosis. As previously discussed, CPPD can present in numerous ways including asymptomatically, in conjunction with osteoarthritis, in the acute phase as pseudogout, or as a type of chronic inflammatory crystal arthritis. Atypical presentations mimicking polymyalgia rheumatica and neuropathic arthropathy have also been described.^3^ As evidenced by the majority of our patients showing a chronic or acute-on-chronic onset, the literature also supports that the associated degenerative arthritis from CPPD causes a gradual onset of pain with slow progressive joint destruction, which is difficult to differentiate from primary osteoarthritis.

Histopathologic and cytologic evaluation of crystalline structures under standard polarized light microscopy for tissue (and compensated polarized light microscopy for synovial fluid) remain the gold standard to diagnose crystalline arthropathies including CPPD.^18^ Calcified deeply basophilic granular aggregates should routinely be subjected to polarization to confirm the presence or absence of refractile crystals. If rhomboid crystals are identified, the surrounding stromal changes, such as chronic inflammation with histiocyte and chondrocyte changes are supportive. In the absence of crystals, amorphous calcifications may likely represent dystrophic calcifications. Key clinical and histopathologic mimics such as other crystal arthropathies (eg gout), infection (eg osteomyelitis, spinal abscess, septic arthritis), and other inflammatory arthropathies (eg rheumatoid arthritis) may be considered in the neuropathologic work up of spinal tissue specimens.

Gouty arthritis of the spine would present as nodules of lightly basophilic fibrillary or feathery collections (tophi) containing needle-shaped MSU crystals that are strongly and negatively birefringent on histology.^19^ Unlike CPPD crystals which remain intact following processing, these MSU crystals do not typically survive processing given their solubility in formalin. If gout is on the differential, then tissue or fluid should be fixed in alcohol to preserve the MSU crystals. If there is an acute flare, neutrophilic infiltrate may be seen. An acute neutrophilic component, or in some cases, a granulomatous reaction also raises the possibility of an infection. With a large collection of neutrophils, a spinal abscess should be considered. Additional findings of devitalized bone and soft tissue necrosis would raise the possibility of acute osteomyelitis. Microorganisms may or may not be present in such cases. Special stains for microorganisms are useful in potentially infectious cases to assess for the presence of fungal hyphae (Gomori methenamine silver or Periodic-acid Schiff stains), bacteria (Gram stain), or mycobacteria (Acid-fast stain: Kinyoun or Ziehl-Neelsen); these findings should be correlated with cultures if taken.^20^ If there is serological or physical evidence of an inflammatory disease like rheumatoid arthritis, histology of spinal cases are reportedly destructive to the bone with chronic inflammatory synovial changes and granulation tissue. The presence of rheumatoid nodules (central fibrinoid necrosis with surrounding palisading granulomatous inflammation) in the spine has been reported.^21^^,^^22^ Evidence of concurrent primary osteoarthritis (OA) is suspected if there is histologic evidence of articular cartilage breakdown (eg fissuring and fragmentation) with chondrocyte proliferation and hypertrophy, calcifications, and subchondral cortical plate thickening.^23^ If OA is identified with crystals, this should prompt a rheumatologic consult to evaluate for an underlying crystal arthropathy. These diagnostic challenges, particularly when CPPD is accompanied by primary osteoarthritis, leads to significant morbidity in patients’ quality of life as they remain untreated for CPPD, perhaps requiring extensive joint instrumentation in an effort to prevent or manage debilitating pain.

While most case reports of axial lumbar CPPD describe those in which the disease process mimics spinal infections, our patient cohort adds to the literature those individuals with claudication and radiculopathy due to lumbar stenosis and degenerative changes, as well as synovial cysts found on imaging and/or intraoperatively. Supported by the literature, we have 3 recommendations to improve the care of these patients to potentially avoid unnecessary invasive procedures and antibiotics that would otherwise contribute to significant morbidity in this vulnerable population.

First, CPPD should be included in the differential for elderly patients with back pain with or without compressive symptoms and non-specific degenerative imaging or findings suspicious for infection. This is particularly relevant when biopsy cultures reveal no identifiable pathogen, and antibiotics introduce the possibility of side effects that may contribute to local drug resistance patterns.

Second, we recommend that spinal tissue sampling for infection and decompression routinely includes evaluation specifically for crystals, especially in patients over 60 years of age with prior trauma or spinal instrumentation. At our institution, all spinal material removed during the described types of procedures was submitted for pathological evaluation, regardless of preoperative suspicion of disease. If tissue is discarded or unexamined, it is unclear how many cases of axial CPPD are undiagnosed in these cases. Furthermore, based on our review, there is at least a small number of patients who are left untreated due to incorrect diagnoses. Therefore, we recommend that tissue be submitted for pathologic analysis even in cases where only a mechanical injury is suspected.

Third, it is highly recommended that when pathological evaluation reveals an entity unsuspected by the primary physician, the pathologist can consider directly contacting the ordering provider. Given that only 2 patients were treated following pathological diagnosis of CPPD, it is possible that pathology reports in cases with low suspicion of impactful diagnoses, such as non-malignant cases or those where patients are not critically ill, are not being routinely reviewed to the extent that may affect management. This could be for a variety of reasons including the time constraints placed on clinicians, difficulty finding the reports within the electronic medical record, difficulty in comprehending the specialized language of pathology reports, or in the case of residents as ordering providers, rotating off service while the specimen is being processed and evaluated. Pathologists routinely contact providers with findings of new malignancies or other critical results but it is possible that adding to that load may be unrealistic given these constraints. As demonstrated by this review, CPPD is a chronic, painful condition that may lead to significant morbidity, such as unnecessary antibiotics and additional surgeries, due to its underrecognition and undertreatment, which adds to the delay of care in this important patient population condition.

One weakness of this study and what presented the authors with the most difficulty was the variation between patient documentation, particularly in terms of temporal profile of presentation and preoperative diagnoses. In a few cases, the preoperative diagnosis on the operative report was not completely inclusive of radiology findings, making it difficult to determine the clinicians’ exact suspicions. To establish consistency, the authors used the preoperative diagnosis as listed in the operative report to define clinical suspicions while also acknowledging that radiology reports may raise different or additional differentials. Delineating between overlap presentations in terms of conditions with similar symptomatology and radiologic findings (an inherent difficulty in making a CPPD diagnosis in general) was another challenge.

Although our study included a wide age range of patients and an even distribution of males and females, it is unlikely that a group of 10 patients is completely representative of the patient population with axial CPPD. However, given its overall rarity and prevalence at our institution that correlates closely with another group’s experience,^7^ as well as the dearth of large studies or case series that focus on CPPD as opposed to all intervertebral disc pathology, our investigation advances this field of study by concentrating on the pathologic features of CPPD and the clinical impact of this disease. In addition, exploration of patients from our single institution included evaluation by multiple neuropathologists, limiting interobserver variability in diagnosis and making it more likely that these 10 patients are indeed the total number of patients with axial CPPD who were operated on at our institution with minimal false negatives. Still, we plan to continue investigating this important area of pathology to more accurately identify these cases and potentially compare them to a larger group of age-matched non-CPPD disc specimens in the future.

It is unclear whether patients who have axial CPPD have additional affected sites. In 2 of our patients who went on to have a rheumatology consult, findings of pre-existing polyarticular arthropathy and radiologic evidence of chondrocalcinosis suggest that the spine was likely not the first site of crystal accumulation in these 2 patients. In the patient who was diagnosed with CPPD with a wrist joint aspiration 4 years after spinal neurosurgical intervention, it remains unknown as to which site was affected first. In the remainder of patients who did not receive a rheumatologic work up but had a favorable outcome, it is impossible to tell if other sites are affected or if they had isolated pathology. As CPPD literature grows, it will be interesting to learn how often axial CPPD is the first presentation of this disease, or if early diagnosis in other joints could possibly save these patients from spinal instrumentation with the use of systemic medications.

Although presentations of CPPD remain less well characterized than traditional MSU crystal arthropathy, one of the most important areas of further investigation that would have the greatest impact on patient outcome is the identification of a disease modifying treatment specific to CPP crystals. At best when diagnosed, patients rely on gout treatments to reduce symptoms of inflammation and pain. This will provide a more definitive treatment and potentially save patients from invasive procedures, whether diagnostic or therapeutic.

It is important to continue reviewing and reporting cases of spinal CPPD to add to the growing literature and to further characterize its presentation. The more often axial CPPD is reported in the literature, the greater its recognition will be. Ultimately, this may lead to earlier diagnosis and more optimal management.

Spinal CPPD is underdiagnosed and undertreated. Together in combination with multidisciplinary care, our recommendations to (1) more often include CPPD in the differential diagnosis of elderly patients with back pain, (2) routinely submit spinal tissue sampling for pathological evaluation, and (3) ensure closed loop communication regarding unsuspected pathology not only have the potential to add to the scarce literature of axial CPPD but also begin a new era of recognition and rhetoric surrounding this diagnosis. This would promote not only prompt identification but also appropriate treatment following clinical and pathological evaluation. Variable clinical presentations and radiographic mimics can be challenging, underscoring the importance of wider recognition of this rare disease among pathologists and involved clinical teams.
